# Protective Effect of Melatonin Against Radiotherapy-Induced Small Intestinal Oxidative Stress: Biochemical Evaluation

**DOI:** 10.3390/medicina55060308

**Published:** 2019-06-25

**Authors:** Ahmed Eleojo Musa, Dheyauldeen Shabeeb, Haider Saadoon Qasim Alhilfi

**Affiliations:** 1 Department of Medical Physics, Tehran University of Medical Sciences (TUMS), International Campus, Tehran 1416753955, Iran; musahmed@yahoo.com; 2 Research Center for Molecular and Cellular Imaging, TUMS, Tehran 1416753955, Iran; 3 Al-Sadder Teaching Hospital, Department of Neurophysiology, Misan 62010, Iraq; 4 University of Misan, Faculty of Medicine, Department of Physiology, Misan 62010, Iraq; 5 University of Misan, Faculty of Medicine, Department of Medicine, Misan 62010, Iraq; dr_yanal72@yahoo.com

**Keywords:** radiotherapy, melatonin, small intestine, oxidative stress, ionizing radiation

## Abstract

*Background and Objectives:* Radiation enteritis is a common side effect after radiotherapy for abdominal and pelvic malignancies. The aim of the present study was to investigate the protective effect of melatonin, known for its free radical scavenging ability, against radiotherapy-induced small intestinal oxidative damage. *Materials and Methods:* Thirty male Wistar rats were randomly assigned to six groups (5 rats in each) as follows: Group I (control group) rats received neither radiation nor melatonin; group II rats received only 8 Gy single dose of gamma radiation to their abdomen and pelvis regions; group III (administered with only 50 mg/kg melatonin); group IV (administered with only 100 mg/kg melatonin); group V (50 mg/kg melatonin + 8 Gy radiation), group VI (100 mg/kg melatonin + 8 Gy radiation). All rats were sacrificed after 5 days for biochemical assessments of their intestinal tissues. *Results:* Treatment with melatonin post irradiation significantly reduced malondialdehyde (MDA) levels as well as increased both superoxide dismutase (SOD) and catalase (CAT) activities of the irradiated intestinal tissues. In addition, melatonin administration with different doses pre irradiation led to protection of the tissues. Moreover, the 100 mg/kg dose was more effective compared to 50 mg/kg. *Conclusions:* The results of our study suggest that melatonin has a potent protective effect against radiotherapy-induced intestinal damage, by decreasing oxidative stress and increasing antioxidant enzymes. We recommend future clinical trials for more insights.

## 1. Introduction

Radiotherapy (RT) is one of the major cancer treatment modalities utilized by approximately 50–70% of cancer patients during their treatment course [1]. RT makes use of ionizing radiation to treat cancers such as abdominal and pelvic malignancies [2]. During RT for abdominal and pelvic malignancies, the intestines are inevitably exposed to radiation. The intestines are highly sensitive to ionizing radiation; hence, radiation-induced intestinal injuries are serious concerns after RT which can lead to a reduction in patient’s quality of life as well as death.

The degree of radiation-induced damages and toxicities to the intestines depends on the radiation dose as well as the volume of intestinal segment that falls within the radiation field [3,4]. Radiation enteritis commonly occurs during RT for abdominal cancers [5]. This complaint is recurrent and produces severe complications [2]. Exposure to ionizing radiation leads to mucosal injury and stimulated inflammatory cells in the gastrointestinal epithelial cells. Furthermore, studies have shown that damages to crypt cells of intestinal epithelium, reduction in the number and sizes of villous structures, as well as ulcers and necrosis, are some of the consequences of exposure to ionizing radiation [6].

Secondary toxicities to the intestinal tract can also occur. Free oxygen radicals (hydroxyl radical (OH), superoxide anion (O_2_–), hydrogen peroxide (H_2_O_2_), etc.), which are produced from the interaction of ionizing radiation with biological tissues, act as cellular mediators to induce intestinal damage [7,8]. Following radiation exposure, elevated levels of free oxygen radicals in the mitochondria cause DNA, protein and lipid damages [9]. As a result, it inhibits replication, transcription and protein synthesis. The intestines have a protective system that prevents oxidative stress or limits its effect. This is mediated by an enzymatic antioxidant system (superoxide dismutase (SOD) and catalase (CAT)), as well as a non-enzymatic antioxidant system (vitamin E and vitamin C) [10]. O_2_– can be neutralized by SOD while H_2_O_2_ can be nullified by catalase (CAT) or glutathione peroxidase (GPx). In light of these findings, several studies related to the use of antioxidants against oxidative damage have been conducted [11,12].

Melatonin (N-acetyl-5-methoxytryptamine), a hormone majorly secreted in the pineal gland, is involved in the circadian regulation of biological and endocrine functions such as mood, sleep, sexual progression and reproduction, immune activities, aging, etc. [13,14,15]. While largely concentrated in several areas of the brain, melatonin has also been observed in various organs and tissues such as the gastrointestinal tract (GIT) [16,17,18] and some leucocytes [19,20]. In plants, melatonin can be found in cereals, olive, walnuts, tomatoes, pineapple, ginger, legumes, etc. [21]. Melatonin has abilities to scavenge free radicals as well as antioxidant effects by stimulating antioxidant enzymes. Furthermore, it has anti-apoptotic and anti-inflammatory effects [22]. Several studies have reported the abilities of melatonin to reduce radiation-induced side effects in various organs such as lens [23], brain [24], liver [25,26], spleen [27], skin [28], lung [29], etc.

Based on these aforementioned properties, the present study aimed to investigate the protective effect of melatonin against radiotherapy-induced small intestinal oxidative damage in rats.

## 2. Materials and Methods

### 2.1. Chemicals

Melatonin was purchased from Sigma Aldrich (St. Louis, MO, USA) while ethanol (5%) was obtained from Tehran Chemie (Tehran, Iran).

### 2.2. Animals

Thirty male Wistar rats (weighing 180–210 g) were purchased from the animal laboratory of Tehran University of Medical Sciences (Tehran, Iran). They were housed under the following conditions: 12 h dark/12 h light cycle (light 8:00 to 20:00 and dark 20:00 to 8:00) to avert the light/dark effect on basal levels of melatonin, 21 ± 1 ˚C room temperature, and 65% humidity. Standard rat diet and water were also provided. The study was approved by the Ethics Committee of the School of Medicine, Tehran University of Medical Sciences (approval number 35116), approved on 3rd of September 2017.

### 2.3. Experimental Design

The animals were randomly divided into six groups (5 rats in each) as follows:

Group I (control group): rats received a one-time normal saline solution (0.9 NaCl) and 5% ethanol via intraperitoneal (IP) administration.

Group II (irradiation group): following anaesthesia with IP administration of ketamine (100 mg/kg) and xylazine (10 mg/kg), a cobalt-60 gamma ray source was used to deliver a single radiation dose of 8 Gy to rats’ abdominal regions, at a source to skin distance (SSD) of 80 cm. Normal saline solution and 5% ethanol was also administered once via IP route.

Group III (melatonin 50 mg/kg): 50 mg/kg melatonin was administered once to the rats via IP route.

Group IV (melatonin 100 mg/kg): 100 mg/kg melatonin was administered once to the rats via IP route.

Group V (melatonin 50 mg/kg + 8 Gy gamma radiation): 50 mg/kg melatonin was administered once via IP route 30 min before irradiation, based on a previous study [30].

Group VI (melatonin 100 mg/kg + 8 Gy gamma radiation): 100 mg/kg melatonin was administered once via IP route 30 min before irradiation, according to previous studies [28,31].

### 2.4. Tissue Samples

Five days after irradiation, all rats were sacrificed. Samples of their small intestines were then collected under sterile conditions. These tissue specimens were frozen in liquid nitrogen, and stored at −35 ˚C. Afterwards, malondialdehyde (MDA) level (a marker of oxidative stress), and antioxidant system markers (SOD and CAT activities) were analysed biochemically.

### 2.5. Biochemical Analysis

100 mg of frozen small intestinal tissues were cut into pieces and homogenized in ice-cold Tris-HCl buffer according to tissue weight (50 mmol/L, pH 7.4) using a homogenizer (Ultra Turrax IKAT18 basic homogenization; Werke, Staufen, Germany) for 3 min at 6000 rpm. The supernatant solution was extracted with an equal volume of ethanol/chloroform mixture (3/5, volume per volume [v/v]). After centrifugation at 3000× *g* rpm for 30 min, the upper layer was used to analyse the total tissue protein levels (MDA, SOD and CAT).

### 2.6. MDA Measurement

MDA levels of the tissue samples were measured using ZellBio MDA kit (ZellBio GmbH, Ulm, Germany). The assay was assessed in terms of the amount of pink colour produced by the interaction of barbituric acid with MDA at high temperature and measured in an acidic media and heat (90–100 °C) as well as at room temperature with the aid of a spectrophotometer (Eon, Bio TeK, Winooski, VT, USA) at 535 nm.

### 2.7. CAT Activity Measurement

CAT activity, representing the amount of sample that will catalyse the decomposition of 1 µmol of H_2_O_2_ to water (H_2_O) and oxygen (O_2_) in 1 min, was measured using ZellBio CAT kit (ZellBio GmbH, Ulm, Germany). The assay was based on the CAT-induced decomposition of H_2_O_2_ into H_2_O and O_2_. A spectrophotometer (Eon, Bio TeK, Winooski, VT, USA) at 405 nm, was used to measure the chromogen colour at room temperature.

### 2.8. SOD Activity Measurement

SOD activity, representing the amount of sample that will catalyse the decomposition of 1 µmol of oxygen radical (O_2_–) to H_2_O_2_ and O_2_ in 1 min, was measured using ZellBio SOD kit (ZellBio GmbH, Ulm, Germany). The conversion of superoxide anion to H_2_O_2_ and O_2_ under enzymatic reaction conditions was applied in this kit. A spectrophotometer (Eon, Bio TeK, Winooski, VT, USA) at 420 nm, was used to measure the chromogen colour at room temperature.

### 2.9. Statistical Analysis

Data were expressed as mean ± standard deviation (SD). All statistical analyses were performed using SPSS software version 22 (IBM, Chicago, IL, USA). One-way analysis of variance (ANOVA) followed by Tukey’s post hoc test, were used to analyse the differences between the various groups. Statistical significance was set at *p* < 0.05.

## 3. Results

### 3.1. MDA Level

**Figure 1:**
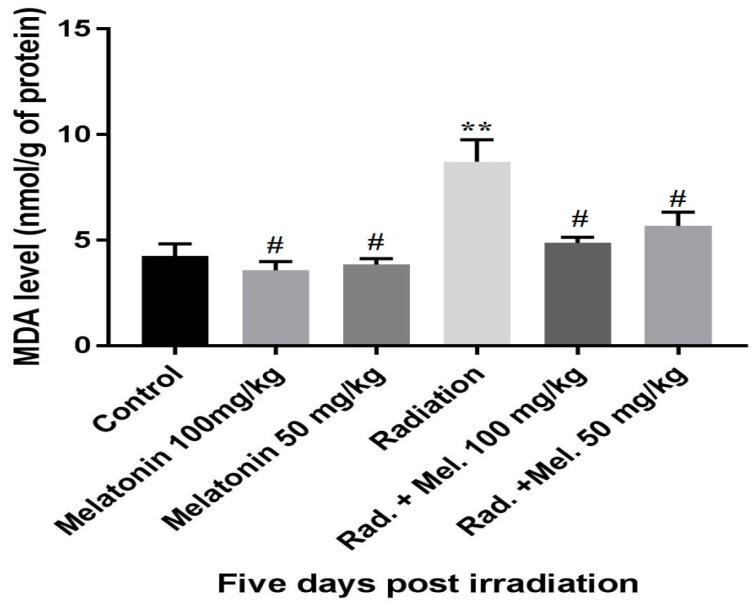
Effects of pretreatments with melatonin (50 and 100 mg/kg) on malondialdehyde (MDA) levels 5 days post irradiation. **Significant difference with control group (*P* < 0.05). ^#^Significant difference with radiation group (*p* < 0.05). Rad. + Mel: signifies the melatonin + radiation group.

Five days after irradiation, MDA levels in the intestinal tissue samples of the radiation treated group were significantly higher compared to both radiation + melatonin (100 mg/kg and 50 mg/kg) and control groups, (*p* < 0.05) (Figure 1). However, treatment with melatonin before irradiation reduced MDA levels significantly (*p* < 0.05). Melatonin also significantly reduced MDA levels in the intestinal tissues compared to control group (*p* < 0.05). No significant differences were observed between the MDA levels of control group compared with radiation + melatonin (50 mg/kg) group (*p* > 0.05). Furthermore, it was observed that the melatonin dose of 100 mg/kg was more effective compared to 50 mg/kg dose in reducing radiation toxicity to the small intestine.

### 3.2. CAT Activity

**Figure 2:**
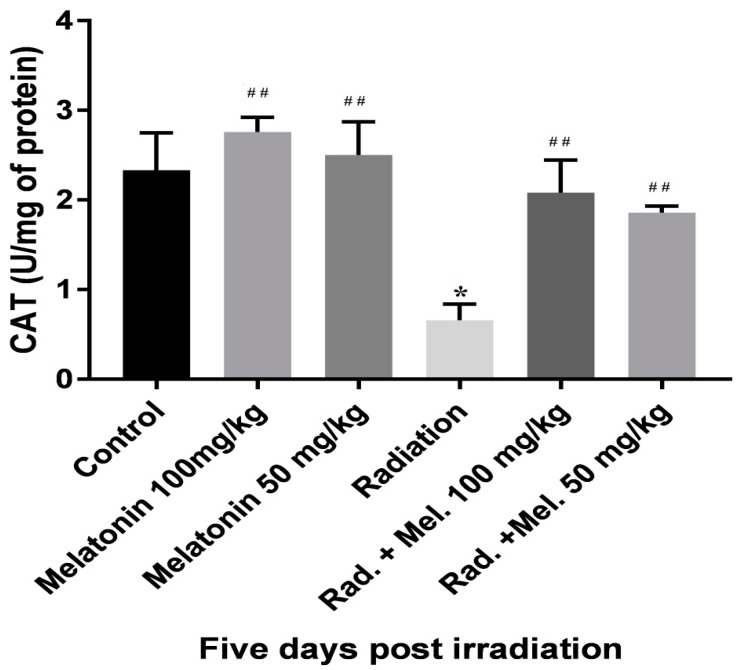
Effect of pretreatments (50 and 100 mg/kg) with melatonin on catalase (CAT) activity 5 days post irradiation. * Significant difference with control group (*p* < 0.05). ^##^ Significant difference with radiation group (*p* < 0.05). Rad. + Mel: signifies the melatonin + radiation group.

Five days after irradiation, results showed significantly lower CAT activity in the small intestinal tissue samples of radiation group compared to control group (*p* < 0.05) (Figure 2). Treatment with melatonin after irradiation reversed CAT activity (*p* < 0.05). In addition, melatonin treatment significantly increased CAT activity of the intestinal tissues compared to control group (*p* < 0.05). No significant difference was observed between the levels of CAT in the intestinal tissues of control group compared with radiation + melatonin (50 mg/kg) group (*p* > 0.05). In addition, 100 mg/kg melatonin dose had a more potent effect compared to 50 mg/kg in reducing radiation-induced toxicity to the small intestine.

### 3.3. SOD Activity

**Figure 3:**
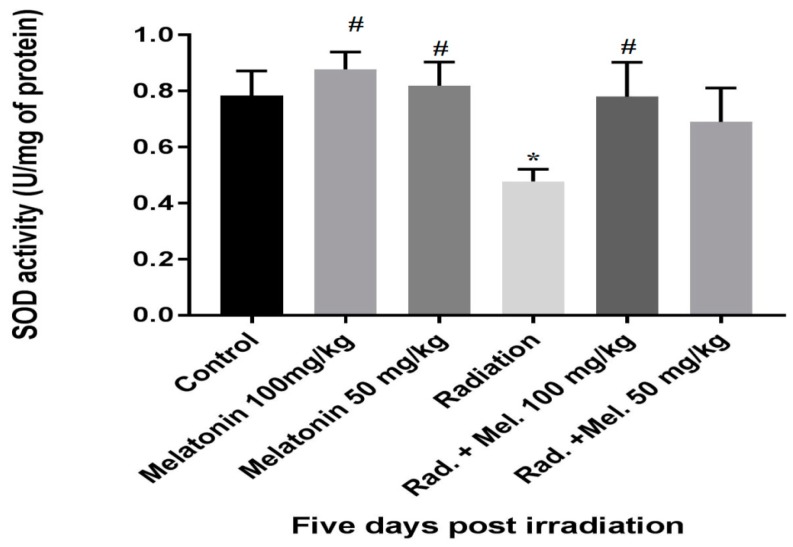
Effect of pre-treatments with melatonin (50 and 100 mg/kg) on superoxide dismutase (SOD) activity 5 days post irradiation. * Significant difference with control group (*p* < 0.05). ^#^ Significant difference with radiation group (*p* < 0.05). Rad. + Mel: signifies the melatonin + radiation group.

As observed in Figure 3, five days after irradiation, there was significantly lower SOD activity in the small intestinal tissue samples of radiation treated group compared to control group (*p* < 0.05). However, treatment with melatonin before irradiation reversed SOD activity to normal (*p* < 0.05). Furthermore, in melatonin (100 mg/kg) group, there was significant increase in the SOD activity of intestinal tissue compared to control group (*p* < 0.05). There was no significant difference between the levels of SOD in the intestinal tissues of control group compared with radiation + melatonin (50 mg/kg) group (*p* > 0.05). Similarly, 100 mg/kg melatonin dose was more effective compared to 50 mg/kg in reducing radiation-induced toxicity to the small intestine.

## 4. Discussion

Small intestinal injury is a severe complication that can arise after RT for abdominal and pelvic cancers. This side effect can affect both surrounding and distant organs [32]. Reactive oxygen species (+) are considered the main underlying cause of intestinal injury [33]. They have been shown to initiate oxidative stress and apoptosis [34]. Studies have suggested that reduction and oxidation (redox) metabolism in cells changes in response to ionizing radiation and plays a central role in radiation toxicity to normal tissue [35]. Free radicals produced after interaction with ionizing radiation cause upregulation of cyclooxygenases (COXs), nitric oxide synthase (NOS), lipoxygenases (LOXs) as well as nicotinamide adenine dinucleotide phosphate oxidase (NADPH oxidase), leading to DNA damage as well as cell death in non-irradiated cells. Mitochondrial functions are also affected, with suppression of mitochondria activity associated with inhibition of ROS/NO production [36]. Advancements in RT methods such as conformal RT, stereotactic body RT (SBRT), intensity-modulated RT (IMRT) and image-guided RT (IGRT) limit the radiation doses to the irradiated volume, thereby sparing healthy tissues during irradiation [37,38]. The Bragg peak phenomenon is also another technique employed in heavy particle radiation for reducing radiation exposure to normal tissues [39].

The use of natural products for protection against detrimental effects of ionizing radiation has been explored in numerous experimental studies [40]. Some of the factors which support the choice of natural products include their minimal toxicities, availability and cost effectiveness [41,42]. Hence, in present study we explored the potentials of a natural agent, melatonin, which has been reported for its potent antioxidant effects against oxidative stress, for protection against radiotherapy-induced small intestinal oxidative injury.

Intestinal toxicity was induced by irradiating the abdomen and pelvis of rats with 8 Gy single dose gamma radiation. Afterwards, we assessed intestinal damage using biochemical parameters. Results of biochemical evaluation showed that exposure to radiation led to significant intestinal tissue damage. Moreover, comparison between the radiation and control groups showed a significant increase in MDA production in the radiation group. Thus, this finding confirms that exposure to radiation induces oxidative stress by increasing MDA levels of the small intestinal tissues [43,44]. MDA is commonly used as a marker of lipid peroxidation in tissues [45]. MDA is secreted as a result of ROS formation due to the oxidation of unsaturated fatty acids in the cell membrane. ROS targets lipids, proteins and DNA [46]. Our results were in agreement with previous studies which showed that the formation of ROS is associated with small intestinal damage [47,48,49].

Another important finding in our study is the reduction in both SOD and CAT activities of small intestinal tissue after radiation exposure. This further demonstrates the adverse effects of radiation on antioxidant system. It has been shown that excess production of free oxygen radicals interrupts the equilibrium between the oxidation and antioxidant systems [50]. This imbalance could result to various diseases, as observed in several studies [28,51]. SOD is a class of antioxidant enzymes with ability to counter cellular oxidative damage due to ROS in the body [52]. During oxidative damage, the level of this enzyme within the tissues is elevated in order to protect them. SOD converts O_2_– into H_2_O_2_ while CAT detoxifies H_2_O_2_ into H_2_O and O_2_ [53].

Furthermore, the current study showed that melatonin treatment (50 mg/kg and 100 mg/kg) before irradiating the small intestinal tissues prevented radiation-induced oxidative stress as well as increased antioxidant system, which are in agreement with a previous study [54]. Moreover, these effects were dose-dependent, with the melatonin dose of 100 mg/kg more effective compared to 50 mg/kg in reducing MDA level and enhancing SOD and CAT activities, which is in agreement with several studies [28,54]. In addition, these melatonin doses had no toxic effect on the animals, which is in line with previous studies that reported the safety of melatonin [55,56].

Several experimental studies have reported different time intervals for observing radiation-induced injury, varying between 3 days to 1 month [50,57]. It has been reported that the initial phase of the effects of ionizing radiation are observed in the first 1–3 days while life threatening effects were observed from 2 weeks after irradiation [58]. Therefore, in present study, we chose the 5-day interval for investigating intestinal damage.

A clinical study by Ben-David et al. reported the ability of melatonin to protect against radiodermatitis (which is commonly observed after RT for breast cancer) [59]. As an adjuvant in chemoradiation for head and neck cancer, melatonin administration has been shown to delay the appearance of grade 3 oral mucositis and grade 2 xerostomia, leading to uninterrupted treatment, thereby improving both treatment outcomes and patients’ quality of life [60,61]. Results from these clinical findings are encouraging towards future clinical studies on the efficacy of melatonin for protection against radiotherapy-induced small intestinal damages.

## 5. Conclusions

In the present study, biochemical evaluations showed that oxidative stress is elevated in radiotherapy-induced small intestinal toxicity. Furthermore, melatonin administration before irradiation improved antioxidant effects by decreasing oxidative stress. Thus, these findings in addition to evidences from literature, suggest that melatonin could prevent the development of enteritis caused by RT. Given the positive effects of melatonin on lipid peroxidation and the antioxidant system in the small intestinal tissue, our findings suggest that it could be an effective radioprotector against radiotherapy-induced small intestinal damages. It is also important to note that the effectiveness of melatonin was dose dependent. We recommend future clinical studies to further assess the efficacy of this natural product in protecting against small intestinal damage due to RT.
